# A positive feedback loop of β-catenin/CCR2 axis promotes regorafenib resistance in colorectal cancer

**DOI:** 10.1038/s41419-019-1906-5

**Published:** 2019-09-09

**Authors:** Baochi Ou, Xi Cheng, Zhuoqing Xu, Chun Chen, Xiaohui Shen, Jingkun Zhao, Aiguo Lu

**Affiliations:** 1 Department of General Surgery, Shanghai Minimally Invasive Surgery Center, Ruijin Hospital, Shanghai Jiao Tong University School of Medicine, Shanghai, 200008 China; 2 Department of General Surgery, Shanghai General Hospital, Shanghai Jiao Tong University School of Medicine, Shanghai, 200025 China

**Keywords:** Oncogenes, Colorectal cancer

## Abstract

Resistance to molecular targeted therapies is a significant challenge for advanced colorectal cancer (CRC). Understanding the underlying mechanisms and developing effective strategies against regorafenib resistance are highly desired in the clinic. Here, we screened the expression of chemokine receptors and identified CC chemokine receptor 2 (CCR2) as a top upregulated gene in regorafenib-resistant cells. CCR2 silencing alleviated drug tolerance in regorafenib-resistant cells, while overexpression of CCR2 enhanced CRC cells resistance to regorafenib. Moreover, CCR2-mediated regorafenib tolerance was demonstrated to be associated with AKT/GSK3β-regulated β-catenin stabilization. In turn, β-catenin modulation is sufficient to trigger the transcriptional activation of CCR2 expression. Clinically, high-CCR2 expression was correlated to shorter overall survival and disease-free survival of patients. A positive correlation between CCR2 and nuclear β-catenin expression was observed in a cohort of CRC tissues. Altogether, these findings suggest β-catenin and CCR2 are part of a positive-feedback loop, which sustains a high CCR2 expression level, conferring CRC cells resistance to regorafenib. Thus, targeting CCR2 may be a useful therapeutic strategy to alleviate regorafenib tolerance to increase the efficacy of CRC treatments.

## Introduction

Colorectal cancer (CRC) is the third leading causes of cancer-related deaths worldwide^1^. In the past 20 years, the treatment for CRC has evolved to the combination of cytotoxic therapy and target-specific vehicles^2^. Current chemotherapeutic regimens utilized in stage IV CRC include fluoropyrimidines, oxaliplatin, irinotecan, and molecular targeted agents (anti-angiogenesis and anti-epidermal growth factor receptor drugs). Despite these impressive advances, recurrence remains common due to the development of drug resistance^3^.

Regorafenib, a multikinase inhibitor targeting the RAS/RAF/MEK/ERK pathway, has been approved to treat metastatic colorectal cancer^4^. Regorafenib inhibits c-Raf, b-Raf, vascular endothelial growth factor receptors (VEGFR), platelet-derived growth factor receptor (PDGFR), and other oncogenic kinases^5^. The antitumor activity of regorafenib has been demonstrated to be correlated with induction of apoptosis, suppression of tumor angiogenesis and proliferation^5^. Although many progressions have been made, the activity of regorafenib is limited by primary and acquired drug resistance. To date, several studies have investigated the mechanisms underlying regorafenib tolerance in human malignancies. It is shown that isomerase Pin1 inhibition reverses the resistance of hepatocellular carcinoma cells to regorafenib^6^. Moreover, antiapoptotic BCL-2 proteins play a key role for regorafenib tolerance in hepatocellular carcinoma^7^. In human CRC, it is reported that FBW7 mutational status mediates cells resistance to regorafenib by blocking Mcl-1 degradation^8^. However, the specific mechanisms in cancer resistance to regorafenib remain unknown.

The Wnt/β-catenin pathway modulates a variety of processes in tumor progression, including cell proliferation, invasion, and metastasis^9^. Recently, it is also reported that Wnt/β-catenin signaling plays a role in cancer resistance to targeted therapies. For instance, the destabilization of Ras overcomes erlotinib tolerance in non-small cell lung cancer through inhibition of Wnt/β-catenin pathway^10^. The study by et al. suggests sorafenib-resistant cells can be eliminated via attenuation of β-catenin signaling^11^. Although Wnt/β-catenin pathway is associated with the effect of regorafenib on tumorigenesis^12^, its function in cancer resistance to regorafenib has not been revealed.

Chemokines are a superfamily of small molecules that are regulated by their interaction with chemokine receptors^13^. Growing evidences have elucidated the critical functions of chemokines and their receptors in tumor biology^14^. Our previous work suggests that CCR4 promotes CRC metastasis via ERK/NF-κB/MMP13 pathway^15^. Moreover, CCR6 facilitates tumor angiogenesis through the AKT/NF-κB/VEGF signaling in colorectal cancer^16^. Recently, the role of ectopic expression of chemokine receptors on cancer cells has been reported to be involved in drug resistance. It is shown that activation of mitogen-activated protein kinase (MAPK) signaling by CXCR7 contributes to enzalutamide resistance in prostate cancer^17^. In esophageal squamous cell carcinoma, cancer-associated fibroblasts derived IL-6 promotes chemoresistance by upregulating CXCR7 expression of tumor cells^18^. Furthermore, CCL2/CCR2 axis is demonstrated to be a contributor to cabazitaxel resistance in prostate cancer cells^19^.

In this study, we hypothesized that chemokine receptors might play important roles in cancer resistance to targeted therapies. Interestingly, the results identified CC chemokine receptor 2 (CCR2) as a top upregulated gene in regorafenib-resistant (regR) cancer cells. Thus, we focused on the function and the underlying mechanism of CCR2 in drug tolerance. We found that CCR2 promoted cells resistance to regorafenib via β-catenin stabilization, and that β-catenin modulation was sufficient to positively regulate CCR2 mRNA and protein expression, by a direct recruitment onto TCF/LEF consensus-binding sites located in CCR2 promoter. Overall, these data suggest targeting CCR2 may be an effective method to alleviate regorafenib resistance, thus increasing the therapeutic efficacy of regorafenib in CRC patients.

## Results

### CCR2 is highly expressed in regorafenib-resistant CRC cells

**Fig. 1:**
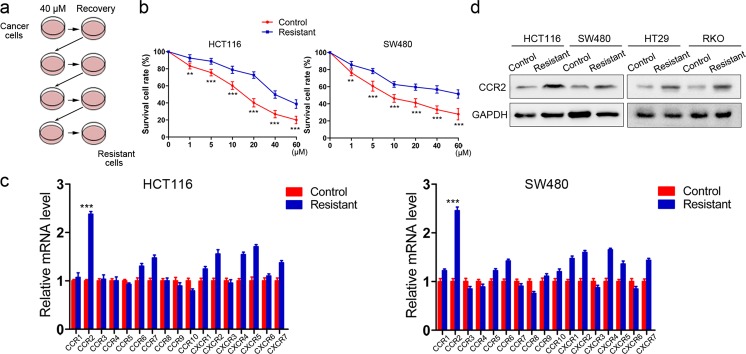
CCR2 expression is highly expressed in regorafenib-resistant cancer cells. **a** Schematic diagram for the establishment of regorafenib-resistant cell lines. **b** CCK-8 assays indicate that resistant cell lines exposed to varying regorafenib doses for 48 h exhibit enhanced tolerance compared to controls. **c** Q-PCR analysis reveals that CCR2 is the most upregulated gene in resistant cells compared with nonresistant controls. **d** Immunoblot analysis shows regorafenib-resistant cells exhibit higher CCR2 expression than control cells. Data represent the mean ± SD and are representative of three independent experiments. **P < 0.01, ***P < 0.001

Primary CRC cell lines (HCT116, SW480) were cultured with regorafenib to generate regR cells (Fig. 1a). To confirm drug tolerance in regR cells, we treated regR and control cell lines with gradient concentrations of regorafenib and compared their viability using a CCK-8 assay (Fig. 1b). We then evaluated whether these regorafenib-resistant cells exhibited changes in expression levels of chemokine receptors. Interestingly, while several receptors were highly expressed in resistant cells relative to nonresistant cells, CCR2 was the most upregulated one (Fig. 1c). This finding was further confirmed in regorafenib-resistant HT29 and RKO cells (Fig. 1d). Thus, we hypothesized that CCR2 played a role in CRC cells tolerance to regorafenib and selected it for further investigation.

### CCR2 plays a critical role in regorafenib tolerance of CRC cells

**Fig. 2:**
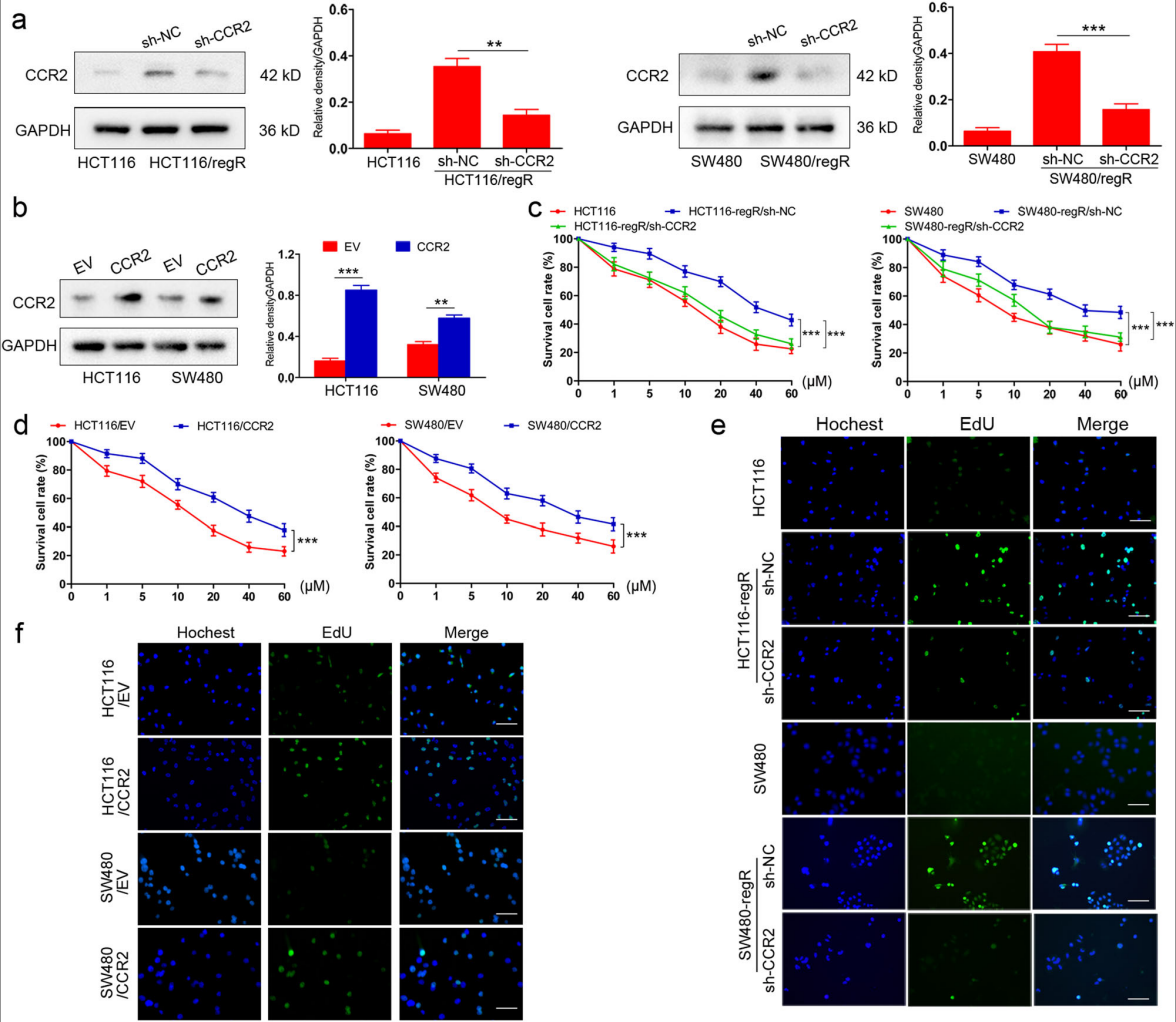
CCR2 is critical for CRC cells resistance to regorafenib. **a** Immunoblot analysis verifying changes in CCR2 expression following shRNA-mediated knockdown. **b** Immunoblot analysis verifying upregulation of CCR2 expression following transfection. EV empty vector. **c** Cell viability of regR/sh-NC and regR/sh-CCR2 cells incubated with gradient doses of regorafenib for 48 h. **d** CCK-8 assay showing ectopic expression of CCR2 enhances cells resistance to regorafenib. **e** EdU staining of regR and regR/sh-CCR2 cells treated with regorafenib (10 μM) for 48 h. Scale, 200 μm. **f** Cell proliferation is promoted by CCR2 overexpression in CRC cells that are cultured with regorafenib (10 μM) for 48 h. Scale, 200 μm. Data represent the mean ± SD and are representative of three independent experiments. *P < 0.05, **P < 0.01, ***P < 0.001

To explore the function of CCR2 in regorafenib resistance of CRC cells, stable clones were generated with either shRNA-mediated CCR2 silencing in regorafenib-resistant cells or a protein ectopic expression in nonresistant cells. The effect of stable transfection was confirmed by western blot (Fig. 2a, b). As shown in Fig. 2c, while regR cells were more tolerant to regorafenib than nonresistant subclones, CCR2 silencing alleviated drug resistance when cells were exposed to gradient doses of regorafenib for 48 h. Furthermore, ectopic expression of CCR2 enhanced HCT116 and SW480 cells resistance to regorafenib (Fig. 2d). In addition, EdU assays showed that regorafenib-treated regR cells were more proliferative than regorafenib-treated nonresistant controls. This effect was decreased upon CCR2 knockdown (Fig. 2e and Supplementary Fig. S1A). Conversely, overexpression of CCR2 could promote cells proliferation ability when they are cultured by regorafenib (10 μM) for 48 h (Fig. 2f and Fig. S1B). Overall, these data indicate that CCR2 plays a critical role in regorafenib tolerance of CRC cells.

### CCR2 promotes β-catenin stabilization through the AKT/GSK3β pathway

**Fig. 3:**
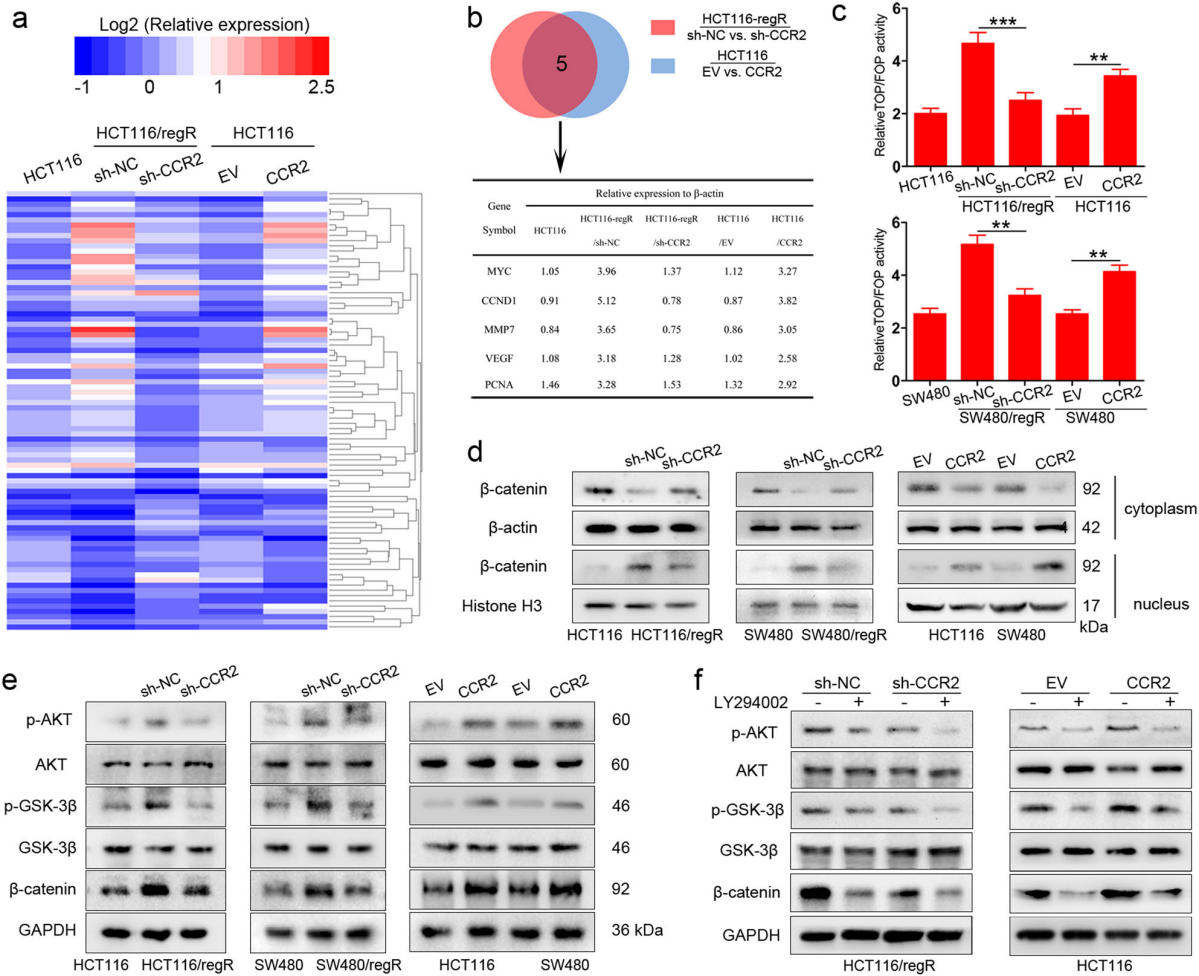
CCR2 stabilizes β-catenin expression through the AKT/GSK3β pathway. **a** A total of 84 genes are quantified using a human signal transduction PCR array. The color scheme represents gene expression changes on a log2 scale. **b** Five genes reach the cutoff value (fold change >2 or <0.5) in both two groups of cells. **c** Silencing of CCR2 reduces TOP/FOP transcription activity in regR cells, while ectopic expression of CCR2 increases TOP/FOP transcription activity of CRC cells. **d** Expression of cytoplasm and nuclear β-catenin in the indicated cell lines, determined by immunoblot. β-actin is used as the cytoplasm protein marker and Histone H3 is used as the nuclear protein marker. **e** Immunoblot analysis of p-ATK (Ser473), AKT, p-GSK3β (Ser9), GSK3β, and β-catenin in the indicated cell lines. **f** Immunoblot analysis of p-ATK (Ser473), AKT, p-GSK3β (Ser9), GSK3β, and β-catenin in the cells treated with AKT pathway inhibitor (LY294002). Data represent the mean ± SD and are representative of three independent experiments. **P < 0.01, ***P < 0.001

To further elucidate the molecular mechanisms of regorafenib tolerance in CRC cells, we performed a signal transduction RT^2^ Profiler PCR Array to profile differentially expressed genes between each group (HCT116-regR/sh-NC vs. HCT116-regR/sh-CCR2 cells, HCT116/EV vs. HCT116/CCR2). The heatmap produced by this array shows gene-expression levels relative to β-actin on a log2 scale (Fig. 3a). Five genes exceeded the cutoff value (fold change >2 or <0.5) in both two groups of cells (Fig. 3b). Among them, MYC, CCND1, MMP7, and VEGF are downstream targets of the β-catenin/T-cell factor (TCF) signaling^20^. We, therefore, hypothesized that CCR2 might regulate regorafenib resistance by targeting β-catenin/TCF signaling. To this end, we conducted a TOP/FOP flash assay and analyzed TOP and FOP luciferase intensity ratios. The results demonstrated that regR cells displayed increased luciferase activity relative to nonresistant cells and this effect could be reduced by CCR2 depletion (Fig. 3c). Conversely, ectopic expression of CCR2 significantly enhanced β-catenin transcriptional activity in CRC cells (Fig. 3c).

β-catenin plays a crucial role through the translocation from the cytoplasm to the nucleus, where it associates with TCF and lymphoid enhancer factor (LEF) to drive the transcription of target genes^9,21^. Thus, we isolated cytoplasm/nuclear protein fractions and analyzed β-catenin expression in different cellular compartments of regR and nonresistant cells. As shown in Fig. 3d and Supplementary Fig. S2a, the inhibition of CCR2 led to an accumulation of β-catenin in the cytoplasm, while ectopic expression of CCR2 enhanced the translocation of β-catenin to the nucleus. To further clarify the mechanisms involved in altering β-catenin expression after changes in CCR2 levels, we screened the effect of CCR2 on other key regulators of β-catenin activation and found AKT/GSK3β signaling might participate in this process. It is known that the phosphorylation of GSK3β at Ser9 inactivates this protein, leading to the inhibition of β-catenin degradation^22^. Moreover, GSK3β could be phosphorylated by AKT signaling to play a role in carcinogenesis^23,24^. Using both regR and non-resistant cells, we observed that ectopic expression of CCR2 increased overall β-catenin level and the expression of p-AKT^Ser473^ and p-GSK3β^Ser9^ (Fig. 3e and Supplementary Fig. S2b). In contrast, silencing CCR2 dramatically decreased the expression levels of p-AKT^Ser473^, p-GSK3β^Ser9^ and β-catenin (Fig. 3e and Supplementary Fig. S2B). Furthermore, LY294002 (the inhibitor against AKT pathway) treatment significantly suppressed the expression of p-AKT^Ser473^, p-GSK3β^Ser9^ and β-catenin in tumor cells (Fig. 3f and Supplementary Fig. S2C). Altogether, these data suggest CCR2 may promote β-catenin stabilization via the AKT/GSK3β signaling in CRC cells.

### CCR2 enhances cells resistance to regorafenib in a β-catenin-dependent manner

**Fig. 4:**
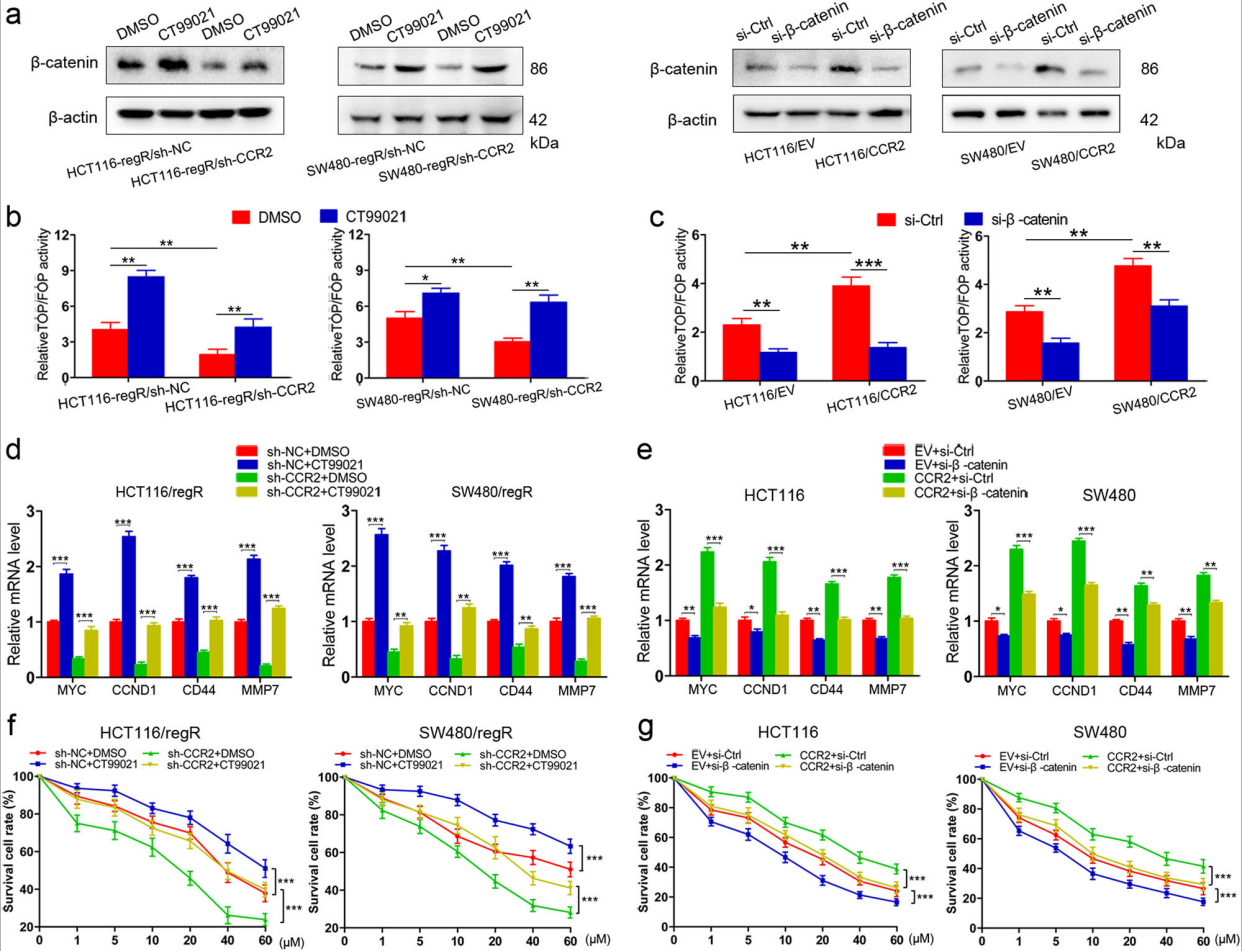
CCR2 promotes cells resistance to regorafenib in a β-catenin-dependent manner. **a** Immunoblot analysis of β-catenin in cells treated with CT99021 or si-β-catenin. **b** TOP/FOP flash assay showing CT99021 increases β-catenin transcriptional activity in both regR/sh-CCR2 and regR/sh-NC cells, while regR/sh-CCR2 cells have lower β-catenin transcriptional activity than regR/sh-NC cells. **c** TOP/FOP flash assay showing si-β-catenin inhibits β-catenin signaling activity in cells transfected with EV or CCR2. **d** The mRNA expression levels of MYC, CCND1, CD44, and MMP7 in regR and regR/sh-CCR2 cells treated with CT99021. **e** Silencing of β-catenin significantly inhibits the mRNA expression of MYC, CCND1, CD44, and MMP7 in cells transfected with EV or CCR2. **f** CT99021 enhances the resistance of regR/sh-NC and regR/sh-CCR2 cells to regorafenib. **g** Inhibition of β-catenin significantly alleviates regorafenib tolerance in CCR2-overexpressing cells and controls. Data represent the mean ± SD and are representative of three independent experiments. *P < 0.05, **P < 0.01, ***P < 0.001

To explore whether β-catenin signaling underlies the function of CCR2 in cells resistance to regorafenib, regR/sh-CCR2 and regR/sh-NC cells were cultured with CT99021, a GSK-3 inhibitor that stabilizes β-catenin expression by protecting it from proteasomal degradation. Furthermore, si-RNA was used to silence β-catenin expression in HCT116/CCR2, SW480/CCR2, and their controls. In fact, both CT99021 and si-RNA caused a significant change in total β-catenin expression relative to the control (Fig. 4a and Supplementary Fig. S3A). TOP/FOP flash assay suggested β-catenin transcriptional activity was significantly improved in both regR/sh-CCR2 and regR/sh-NC cells in response to CT99021, while regR/sh-CCR2 cells were found to have lower β-catenin transcriptional activity than regR/sh-NC cells (Fig. 4b). Moreover, while CCR2-overexpressing cells had higher β-catenin transcriptional activity than control cells, the si-β-catenin was still able to significantly suppress this effect in both groups of cells (Fig. 4c).

We further assessed the effect of β-catenin alterations on the expression of downstream target genes (MYC, CCND1, CD44, and MMP7) in regR/sh-CCR2, CCR2-overexpressing cells and their controls. The results demonstrated that CT99021 increased the expression levels of downstream genes in both regR/sh-CCR2 and regR/sh-NC cells (Fig. 4d). Moreover, compared with the si-Ctrl, silencing of β-catenin significantly decreased the mRNA levels of target genes in CRC cells transfected with EV or CCR2 (Fig. 4e). In addition, CCK-8 assays showed that CT99021 could enhance the resistance of both regR/sh-NC and regR/sh-CCR2 cells to regorafenib (Fig. 4f). Conversely, the inhibition of β-catenin significantly alleviated regorafenib tolerance in CCR2-overexpressing cells and controls (Fig. 4g). Overall, our data demonstrate that CCR2 promotes cells resistance to regorafenib in a β-catenin-dependent manner.

### β-catenin is a direct transcriptional activator of CCR2 expression

Next, we investigated the mechanism responsible for the upregulation of CCR2 in regorafenib-resistant cells. As revealed by above-mentioned PCR array, the β-catenin signaling was significantly activated in HCT116/regR cells versus non-resistant controls (Fig. 3b). Moreover, there are two TCF/LEF consensus-binding sites distributed along the functional promoter of CCR2. Considering TCF/LEF proteins are known as the β-catenin co-factors, we assumed that CCR2 expression might be transcriptionally regulated by β-catenin.

**Fig. 5:**
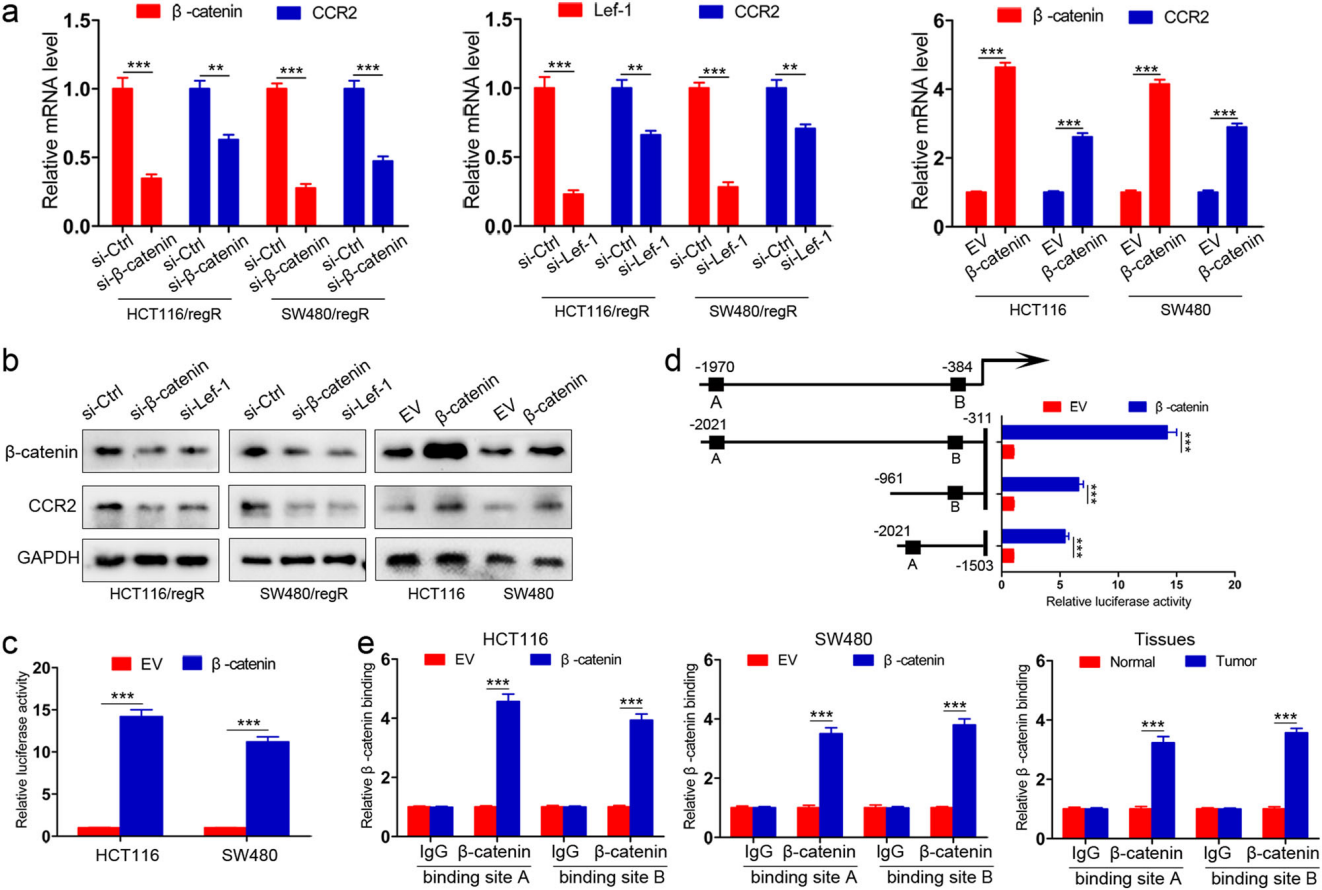
CCR2 expression is transcriptionally regulated by β-catenin. **a** The mRNA expression of β-catenin, CCR2 or Lef-1 in cells transfected with si-β-catenin, si-Lef-1 or β-catenin plasmids. **b** Immunoblot analysis of β-catenin and CCR2 in cells with inhibition of β-catenin and Lef-1, or β-catenin overexpression. **c** The CCR2 promoter luciferase construct (−2021/−311) CCR2 was cotransfected with pGL3-β-catenin, and promoter activities were detected in HCT116 and SW480 cells. **d** Selective deletion analyses identified both sites A and B were β-catenin-responsive regions in the CCR2 promoter. The schematic constructs are shown (left), and the bar graphs present the relative levels of luciferase activity (right). **e** A ChIP assay demonstrated the direct binding of β-catenin to TCF/LEF consensus-binding sites on the CCR2 promoter in CRC cells and tissues. Data represent the mean ± SD and are representative of three independent experiments. **P < 0.01, ***P < 0.001

We first tested the effect of β-catenin knockdown in regorafenib-resistant cells, where CCR2 is highly expressed. We found that an efficient silencing of β-catenin led to a significant decrease of CCR2 mRNA expression (Fig. 5a). As β-catenin acts in association with TCF/LEF transcription factors, we also used siRNA to silence Lef-1 gene and observed a similar reduction in CCR2 mRNA levels (Fig. 5a). Moreover, the downregulation of CCR2 transcripts translated into a strong decline of the protein level (Fig. 5b and Supplementary Fig. S3B). On the contrary, ectopic expression of β-catenin in CRC cell lines was sufficient to turn on CCR2 gene expression at the mRNA (Fig. 5a) and protein (Fig. 5b and Supplementary Fig. S3B) levels.

We then explored whether β-catenin regulates CCR2 expression directly, through the two putative TCF/LEF consensus-binding sites found in CCR2 promoter. These sites are located in upstream (A, −1970bp; B, −384bp) relative to the transcription start site of CCR2. A luciferase reporter assay showed that β-catenin transactivated CCR2 promoter activity (Fig. 5c) in HCT116 and SW480 cells. Sequence analysis showed that both two binding sites were critical for β-catenin-induced CCR2 transactivation (Fig. 5c). A chromatin immunoprecipitation assay further confirmed the enrichment of β-catenin on the TCF/LEF consensus sites of CCR2 promoter in CRC cells and human CRC tissues (Fig. 5d). Thus, β-catenin is directly recruited onto the TCF/LEF consensus-binding sites on CCR2 promoter, leading to CCR2 expression activation in CRC cells.

### Upregulation of CCR2 confers regorafenib-tolerance traits to CRC cells

**Fig. 6:**
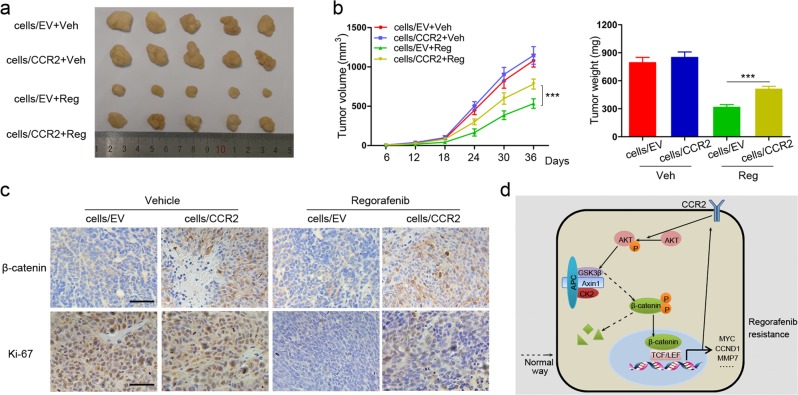
Upregulation of CCR2 confers regorafenib resistance to CRC cells in vivo. **a** Representative images of xenografts formed by injection of nude mice with tumor cells, followed by regorafenib (Reg) or vehicle (Veh) treatment. **b** Growth curves and average weight of subcutaneous xenografts formed in nude mice. **c** IHC staining of β-catenin and Ki-67 in the tissues of xenografts. Scale, 50 μm. **d** Schematic illustration of the mechanism by which CCR2 promotes CRC cells resistance to regorafenib

The preceding data raised a question concerning whether upregulation of CCR2 could sufficiently promote regorafenib resistance in vivo. To this end, the tumor cells were subcutaneously injected into nude mice to establish a xenograft model. Following tumor generation, we treated these mice with regorafenib or vehicle. The results showed the xenografts derived from CCR2-overexpressing cells exhibited increased volume and weight than that from controls in response to regorafenib (Fig. 6a, b). Moreover, we validated that β-catenin expression was upregulated in CCR2-overexpressing xenografts (Fig. 6c). Ki-67 expression was also significantly improved in CCR2-overexpressing xenografts (Fig. 6c), suggesting a higher proliferative ability of CCR2-overexpressing cells under drug stress. This confirmed that overexpression of CCR2 could confer regorafenib resistance to CRC cells in vivo. Altogether, the schematic diagram of our study is shown in Fig. 6d.

### Clinical significance of CCR2 and nuclear β-catenin expression in CRC specimens

**Fig. 7:**
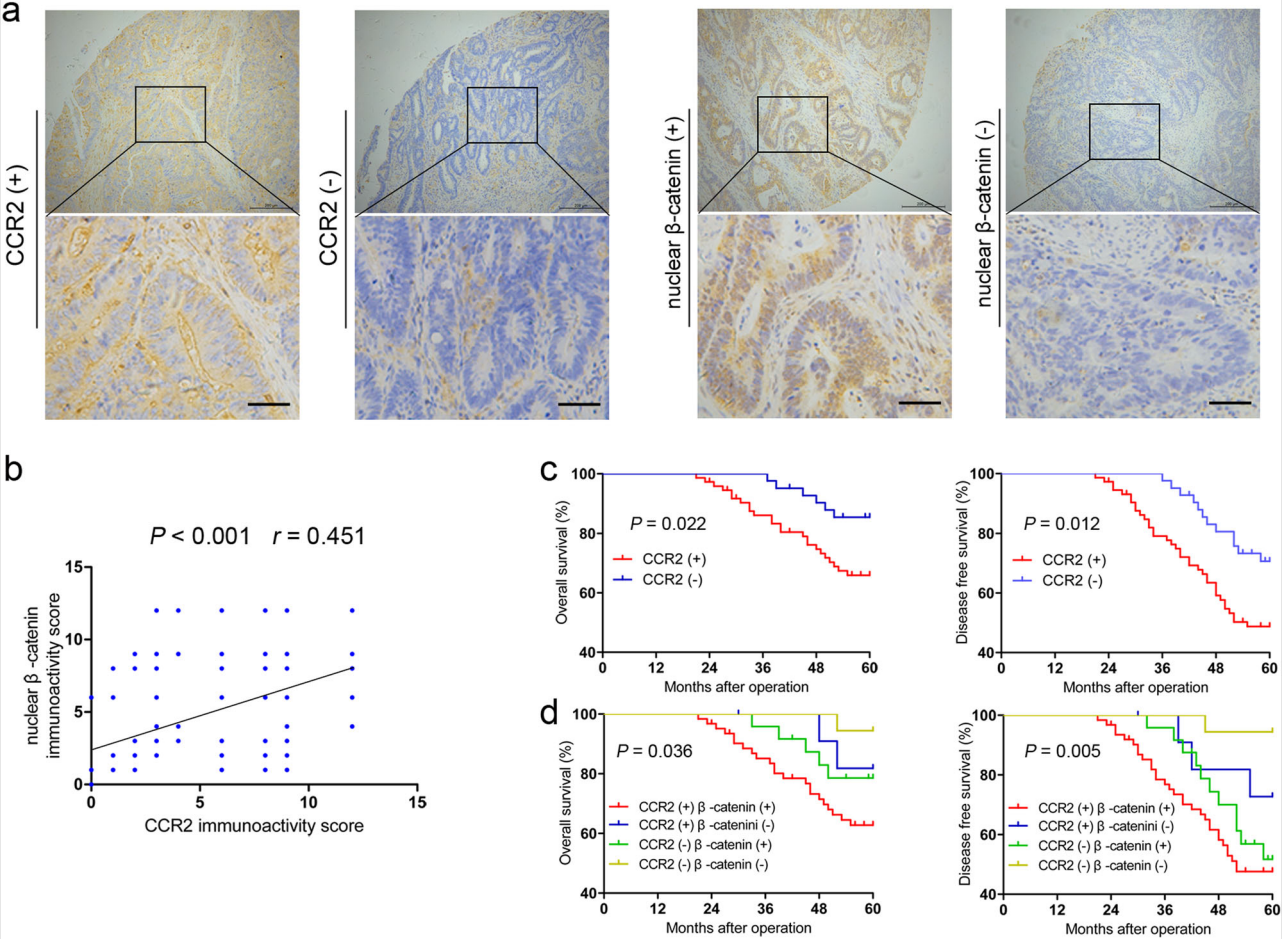
Clinical significance of CCR2 and nuclear β-catenin expression in CRC patients. **a** Representative images of CCR2 and nuclear β-catenin staining in a cohort of 116 CRC tissues. Scale, 50 μm. **b** The correlation between CCR2 and β-catenin expression was analyzed in a cohort of 116 CRC tissues (Pearson’s correlation, *P* < 0.001, *r* = 0.451). **c** Patients with positive expression of CCR2 presented with worse overall survival, and disease-free survival compared with that of negative expression of CCR2. **d** Prognostic values of CCR2 combined with β-catenin

To investigate the clinical significance of CCR2 and nuclear β-catenin, we detected their expression levels using a previously reported tissue microarray^25^. Representative images of CCR2 and nuclear β-catenin staining are shown in Fig. 7a. Notably, out of the 74 CCR2^positive^ cases, 62 (83.8%) displayed prominent nuclear β-catenin expression. Meanwhile, of the 42 CCR2^negative^ cases, 18 (42.9%) were nuclear β-catenin negative (*P* *=* 0.002, Supplementary Table S1). Pearson’s correlation analysis indicated that CCR2 expression was positively correlated with nuclear β-catenin expression (Fig. 7b, *P* < 0.001, *r* = 0.451) in this cohort. Patients in the CCR2^positive^ group had a significantly poorer overall survival than those in the CCR2^negative^ group (Fig. 7c), which was consistent with previous finding^26^. Moreover, CCR2^positive^ patients had a shorter disease-free survival (Fig. 7c). Interestingly, the patients whose tumors expressing high levels of CCR2 and nuclear β-catenin exhibited worst prognoses (Fig. 7d). Multivariate analysis indicated that, however, CCR2 expression was not an independent prognostic risk factor (data not shown).

## Discussion

Incorporation of molecular targeted agents has been shown to improve efficacy of CRC treatment, however, some patients exhibit heterogeneous responses and eventually become resistant^3^. Thus, uncovering the mechanism underlying drug resistance in colorectal cancer is essential to optimizing therapeutic strategies. Chemokines and their receptors are initially characterized due to their orchestration of cell migration and homing in health and disease. Moreover, the chemokine superfamily has been involved in various biological processes such as inflammatory mobilization of leukocytes, angiogenesis, wounds healing, and cancer metastasis^27^. However, the role of chemokine receptors during the process of targeted therapies failure remains unknown.

In the present study, we used common CRC cell lines that were selected over time for acquired resistance to regorafenib. While multiple mechanisms of regorafenib resistance likely operate in these cells, we focused on chemokine receptors. After weeks of exposure to increasing concentrations of drugs, we observed remarkably elevated CCR2 levels in regorafenib-resistant cells compared to control group, suggesting this protein contributes to cell survival in the presence of regorafenib. Thus, our study aimed to explore the function of CCR2 in the development of regorafenib tolerance.

As an important chemokine receptor, CCR2 is mainly recruited to the sites of inflammation by CCL2 in various cell types, including myeloid cells, monocytes, and macrophages^28–31^. The function of CCR2 has been demonstrated to participate in several processes of cancer progression. In breast cancer, CCL2/CCR2 axis coordinates cells survival and motility through Smad3 and MAPK-dependent mechanisms^32^. Moreover, colon cancer cell-derived CCL2 activates CCR2 on endothelial cells, thereby enabling efficient cancer cell extravasation^33^. In this study, we demonstrated that CCR2 silencing attenuated drug tolerance of regorafenib-resistant cells when they were exposed to varying doses of drug. EdU assays suggested CCR2 knockdown decreased the proliferative capacity of regR cells following regorafenib treatment. Conversely, ectopic expression of CCR2 enhanced CRC cells resistance to regorafenib. Indeed, our in vivo investigation also found that CCR2-overexpressing cells exhibited a poor response to regorafenib treatment.

To investigate the molecular mechanisms responsible for regorafenib tolerance in CRC cells, we used a PCR array to profile differentially expressed signal transduction-related genes in two groups of cells (HCT116-regR/sh-NC vs. HCT116-regR/sh-CCR2 cells, HCT116/EV vs. HCT116/CCR2). Our results suggested β-catenin/TCF signaling might play a crucial role in CCR2-mediated drug resistance. To date, β-catenin signaling has been demonstrated to be critical for maintaining chemoresistance in tumor cells^34^. For instance, PHF19 activates β-catenin signaling in glioblastoma to confer doxorubicin resistance^35^. In CRC, the hypermethylation of MEIS2 is associated with oxaliplatin-based chemotherapy tolerance^36^. Herein, using the TOP/FOP flash assay, we found that CCR2 depletion could reduce the β-catenin transcriptional activity of regR cells, while ectopic expression of CCR2 significantly enhanced the transcriptional activity in CRC cells. Furthermore, β-catenin expression was analyzed in different cellular compartments of tumor cells. The results suggested CCR2 silencing led to an accumulation of β-catenin in the cytoplasm, while ectopic expression of CCR2 enhanced the translocation of β-catenin to the nucleus. GSK3β is a critical component of the destruction complex that facilitates the phosphorylation and degradation of β-catenin^22^. The function of GSK3β could be suppressed by p-ATK via the phosphorylation of serine residue^37^. Indeed, the expression levels of p-AKT^Ser473^, p-GSK3β^Ser9^ and total β-catenin were all significantly changed by silencing or overexpression of CCR2. Moreover, when treated with AKT pathway inhibitors (LY294002), the expression of p-GSK3β^Ser9^ and β-catenin was significantly blocked in tumor cells. Thus, β-catenin could be stabilized by CCR2 through the AKT/GSK3β pathway in CRC cells.

We then investigated whether β-catenin stabilization regulated by CCR2 could contribute to regorafenib resistance. CT99021 was used to restore β-catenin expression in CCR2-silencing regR cells, while si-RNA was used to knockdown β-catenin in CCR2-overexpressiong CRC cells. Interestingly, CT99021 treatment in regR cells with CCR2 silencing led to the activation of β-catenin transcriptional activity, upregulation of downstream target genes and promotion of cells tolerance to regorafenib. Conversely, the inhibition of β-catenin in CRC cells with CCR2 overexpression resulted in the inactivation of β-catenin transcriptional activity, downregulation of downstream target genes and alleviation of regorafenib resistance.

The preceding findings promoted us to explore the mechanism for the upregulation of CCR2 in regorafenib-resistant cells. First, we found that silencing of β-catenin or Lef-1 led to a significant decrease of CCR2 mRNA and protein levels. On the contrary, ectopic expression of β-catenin was sufficient to turn on CCR2 gene expression levels. Subsequent luciferase reporter assay suggested β-catenin transactivated CCR2 promoter activity in HCT116 and SW480 cells. Moreover, both binding sites A and B were demonstrated to be critical for β-catenin-induced CCR2 activation. A ChIP assay further confirmed the enrichment of β-catenin on CCR2 promoter in CRC cells and tissues. Thus, β-catenin is directly recruited onto the TCF/LEF consensus-binding sites located in CCR2 promoter, leading to CCR2 expression activation. Clinically, IHC analysis revealed a positive correlation between CCR2 and nuclear β-catenin expression in a cohort of tissues. High expression of CCR2 in CRC tissues was significantly associated with shorter overall survival and disease-free survival. Furthermore, combination of CCR2 and nuclear β-catenin was a more powerful prognostic marker for patients with CRC.

In conclusion, this study identifies CCR2 as a top upregulated chemokine receptor in regorafenib-resistant cancer cells. Moreover, β-catenin and CCR2 are part of a positive feedback loop, which sustains a high CCR2 expression level, conferring CRC cells resistance to regorafenib. Our findings thus identify new roles for CCR2 in the development of regorafenib tolerance and suggest that CCR2 may be a potential target for overcoming CRC drug resistance.

## Materials and methods

### Cells and reagents

CRC cells (HCT116, SW480, HT29, and RKO) were purchased from the ATCC (Rockville, MD) and authenticated by STR/DNA profiling by BIOWING BIOTECHNOLOGY (Shanghai, China). Cells were cultured in RPMI 1640 media containing 10% FBS at 37 °C and confirmed as mycoplasma free using a Mycoplasma Detection Kit (Roche). Stable resistant cells were generated as previously described^8^. Briefly, cancer cells were cultured at a concentration of 40 μM regorafenib for 3 days, followed by recovery for 5 days. Then the period of treatment and recovery was repeated for a total of 4 cycles. Regorafenib, LY294002 (PI3K/AKT pathway inhibitor) and CT99021 (GSK-3 inhibitor that stabilizes β-catenin expression) were purchased from Selleck Chemicals (USA) and used according to manufacturer’s instructions.

### Quantitative reverse transcription polymerase chain reaction (q-PCR) and PCR array

Total RNA was extracted using TRIzol reagent. We then used a reverse transcription kit (Invitrogen) to perform reverse transcription. Finally, q-PCR was performed in an Applied Biosystems 7500 System with primers, mixture of cDNA and SYBR Green PCR Master Mix (Applied Biosystems). In addition, the Human Signal Transduction PathwayFinder RT^2^ Profiler PCR Array (QIAGEN) was conducted according to the manufacturer’s instructions. Target gene expressions were compared to β-actin as a control. The sequences of primers were depicted in Supplementary Table S2.

### Immunoblotting

Nuclear proteins were extracted using NE-PER Nuclear and Cytoplasmic Extraction Reagents (Thermo Scientific). Protein lysates were collected from cells in RIPA buffer with 1% protease inhibitor, and protein lysate concentration was measured by bicinchonininc acid. Proteins were detected following an overnight incubation at 4 °C with primary antibodies, followed by 2 h incubation with horseradish peroxidase-conjugated secondary antibodies at room temperature. A LI-COR Odyssey Infrared fluorescence scanner was used to capture the images. Antibodies against CCR2 (12199), β-catenin (8480), β-actin (4970), Histone H3 (4499), AKT (40D4), p-Ser473-AKT (4060), and p-Ser9-GSK3β (9322) were purchased from Cell Signaling Technology. Antibodies against GAPDH (ab181602) and GSK3β (ab32391) were purchased from Abcam.

### Lentiviral transfection and siRNA knockdown

The short hairpin RNA (shRNA) targeting CCR2 and pGL3-CCR2 lentivirus vector were established by Genepharma (China). Tumor cells were seeded onto six-well plates at 3 × 10^5^ cells per well. When the cell density reached about 70%, lentivirus particles were transfected into cells in the presence of polybrene. Stably transfected cells were selected with 5 μg/ml puromycin and tested regularly by immunoblotting to ensure downregulation or upregulation of CCR2. In some experiments, cells were transiently transfected with β-catenin or Lef-1 siRNA (Genepharma, China) using Lipo3000 (Invitrogen) according to the manufacturer’s instructions.

### Immunohistochemistry (IHC)

The tissue microarray used in this study has been previously reported^25^. All protocols were authorized by the Ethics Committee of Shanghai Ruijin Hospital. Tissue staining was performed as previously described^25^. Ten randomly chosen fields were captured for each slide. The staining score was independently evaluated by two pathologists according to the percentage of positive cells and staining intensity. Notably, the evaluation of nuclear β-catenin was based on percentage of positive nucleus and intensity of nucleus. Scoring for percentage was: 0% (0), 1–5% (1), 6–29% (2), 30–59% (3), and more than 60% (4). Scoring for staining intensity was: no staining (0), slight staining (1), moderate staining (2), and intense staining (3). These values were multiplied together to provide a score for each case. Cases were grouped as either negative (score 0–4) or positive (score >4) for analysis.

### CCK-8 assay and EdU labeling

Cells were seeded in a 96-well plate overnight at a density of 5 × 10^3^ cells per well and treated with the indicated doses of regorafenib (1, 5, 10, 20, 40, 60 µM) for 48 h. Then, the cells were incubated with 10 µL CCK-8 for 60 min at 37 °C, 5% CO_2_. The absorption value was detected at 450 nm with a spectrophotometer. Each assay was conducted in triplicate and repeated three times. In addition, cell proliferation rates were analyzed by measuring DNA synthesis using the Click-iT EdU Assay (Roche, USA).

### Nude mouse xenograft models

Four-week-old male nude BALB/C mice were subcutaneously injected with CCR2-overexpressing or control cells (*n* = 10 per group). When tumor volumes reached approximately 0.2 cm^3^, mice were intravenously treated vehicle or 45 mg/kg regorafenib (orally) twice a week for three weeks. Body weights and tumor volumes were measured every other day. Tumor volume was calculated using the formula: length × width^2^ × 0.5. All experiments were performed according to the official recommendations of the Chinese animal community.

### TOP/FOP flash reporter assay

Tumor cells were seeded in 24-well plates and transfected with 2 ng of pRL-TK Renilla luciferase vector and either TOP flash plasmid or FOP flash plasmid (200 ng) using Lipofectamine 3000. Forty-eight hour later, luciferase activity was measured using the Dual Luciferase Reporter Assay (Promega, USA) according to the manufacturer’s protocol. The results were presented as the relative TOP/FOP value.

### Luciferase assay

CCR2 promoter fragments were amplified from human genomic DNA, and were inserted into pGL3-basic vector. The β-catenin-overexpressing or control cells were cotransfected with pGL3-CCR2-promotor constructs. Luciferase assays were performed 24 h after transfection using Dual-Luciferase Reporter Assay System (Promega) following the manufacturer’s instructions. The ratios of luminescence intensities were measured relative to that of the pGL3 empty vector. The primers are presented in Supplementary Table S3.

### Chromatin immunoprecipitation (ChIP)

ChIP assay was performed as previously described^15^. Briefly, after crosslinking with 1% formaldehyde, protein and DNA were extracted by SDS lysis buffer and sheared by sonication. The supernatants were incubated overnight at 4 °C with 2 µg anti-β-catenin (#8480, Cell Signaling Technology) or control rabbit immunoglobulins. After purification of precipitated DNA fragments, q-PCR was performed using the listed primers (Supplementary Table S4).

### Statistical analysis

All statistics were expressed as mean ± SD and were analyzed using SAS 9.3 (SAS Institute Inc., USA). All experiments were conducted at least three times. ANOVA and Student’s t test were performed to compare differences among experimental groups. *P* < 0.05 was considered statistically significant.

## Supplementary information

Supplementary Table.

Figure S1

Figure S2

Figure S3

Supplementary figure legends.
