# Supplementary material for: A positive feedback loop of β-catenin/CCR2 axis promotes regorafenib resistance in colorectal cancer
Source: Cell Death Dis. 2019 Sep 9;10(9):643. doi: 10.1038/s41419-019-1906-5 (PMC6733926; doi:10.1038/s41419-019-1906-5)
Supplement: Supplementary file 1 — Supplementary Table. [file 41419_2019_1906_MOESM1_ESM.doc]

**Table S1** The primers used in this study.

| Primers used for Q-PCR: | | |
| --- | --- | --- |
| CCR1 forward | | GACTATGACACGACCACAGAGT |
| CCR1 reverse | | CCAACCAGGCCAATGACAAATA |
| CCR2 forward | | TACGGTGCTCCCTGTCATAAA |
| CCR2 reverse | | TAAGATGAGGACGACCAGCAT |
| CCR3 forward | | TGGCATGTGTAAGCTCCTCTC |
| CCR3 reverse | | CCTGTCGATTGTCAGCAGGATTA |
| CCR4 forward | | CCCACGGATATAGCAGACACC |
| CCR4 reverse | | GTGCAAGGCTTGGGGATACT |
| CCR5 forward | | TTGCCAAACGCTTCTGCAAAT |
| CCR5 reverse | | AGTGGATCGGGTGTAAACTGA |
| CCR6 forward | | TTCAGCGATGTTTTCGACTCC |
| CCR6 reverse | | GCAATCGGTACAAATAGCCTGG |
| CCR7 forward | | TGAGGTCACGGACGATTACAT |
| CCR7 reverse | | GTAGGCCCACGAAACAAATGAT |
| CCR8 forward | | CTGTCTGACCTGCTTTTTGTCT |
| CCR8 reverse | | CCACTTTGCACATTACAGTCCC |
| CCR9 forward | | ATGTCAGGCAGTTTGCGAG |
| CCR9 reverse | | TGCAGTACCAGTAGACAAGGAT |
| CCR10 forward | | TGAAGAGGACGCATACTCGG |
| CCR10 reverse | | CCACGGTCAGGGAGACACT |
| CXCR1 forward | | CTGACCCAGAAGCGTCACTTG |
| CXCR1 reverse | | CCAGGACCTCATAGCAAACTG |
| CXCR2 forward | | CCTGTCTTACTTTTCCGAAGGAC |
| CXCR2 reverse | | TTGCTGTATTGTTGCCCATGT |
| CXCR3 forward | | CCACCTAGCTGTAGCAGACAC |
| CXCR3 reverse | | AGGGCTCCTGCGTAGAAGTT |
| CXCR4 forward | | ACTACACCGAGGAAATGGGCT |
| CXCR4 reverse | | CCCACAATGCCAGTTAAGAAGA |
| CXCR5 forward | | CACGTTGCACCTTCTCCCAA |
| CXCR5 reverse | | GGAATCCCGCCACATGGTAG |
| CXCR6 forward | | TCCTGGTGAACCTACCCCTG |
| CXCR6 reverse | | AAACACCCATTCATGGATGCC |
| CXCR7 forward | | TCTGCATCTCTTCGACTACTCA |
| CXCR7 reverse | | GTAGAGCAGGACGCTTTTGTT |
| β-actin forward | | CATGTACGTTGCTATCCAGGC |
| β-actin reverse | | CTCCTTAATGTCACGCACGAT |
| MYC forward | | GGCTCCTGGCAAAAGGTCA |
| MYC reverse | | CTGCGTAGTTGTGCTGATGT |
| CCND1 forward | | GCTGCGAAGTGGAAACCATC |
| CCND1 reverse | | CCTCCTTCTGCACACATTTGAA |
| CD44 forward | | CTGCCGCTTTGCAGGTGTA |
| CD44 reverse | CATTGTGGGCAAGGTGCTATT | |
| MMP7 forward | GAGTGAGCTACAGTGGGAACA | |
| MMP7 reverse | CTATGACGCGGGAGTTTAACAT | |

**Table S2.** Primers for construction of luciferase reporter plasmid.

| Primers for CCR2 promoter construct: | Primer sequences | Enzyme |
| --- | --- | --- |
| (-2021/-311) CCR2 sense: | GAGGTGCCCTCTGTTATCCTGCCCTCT | BspLI |
| (-961/-311)CCL2 sense: | GAGGGGCCAACTTAGATGACACCATGT | BspLI |
| Antisense: | GGGAATTCATTATTAACTACCGTCACCC | EcoRI |
| (-2021/-1503) CCR2 sense: | GAGGTGCCCTCTGTTATCCTGCCCTCT | BspLI |
| Antisense: | TATACCCGGGCTGGTCTCGAACTCCTG | AvaI |

**Table S3.** Primers for PCR in chromatin immunoprecipitation.

| Primer | | sequence | position | product |
| --- | --- | --- | --- | --- |
| Primer A | F | GGCTCTGCAACTATCTACCT | -2029bp | 112bp |
|  | R | TGCTCTGGCTGATTAGGA | -1917bp |  |
| Primer B | F | AGAGGAGGTGAGAATGGG | -623bp | 369bp |
|  | R | TGTGCTGGTTTCAGTGGT | -254bp |  |

**Table S4.** The correlation between CCR2 and β-catenin in tumor invasion and lymphatic metastatic cases of CRC as judged by immunohistochemistry.

|  | CCR2 (+) | CCR2 (-) | *P* value |
| --- | --- | --- | --- |
| β-catenin (+) | 62 | 24 | 0.002 |
| β-catenin (-) | 12 | 18 |
| Cases | 74 | 42 |
