# Supplementary figures and images for: A positive feedback loop of β-catenin/CCR2 axis promotes regorafenib resistance in colorectal cancer
Source: Cell Death Dis. 2019 Sep 9;10(9):643. doi: 10.1038/s41419-019-1906-5 (PMC6733926; doi:10.1038/s41419-019-1906-5)

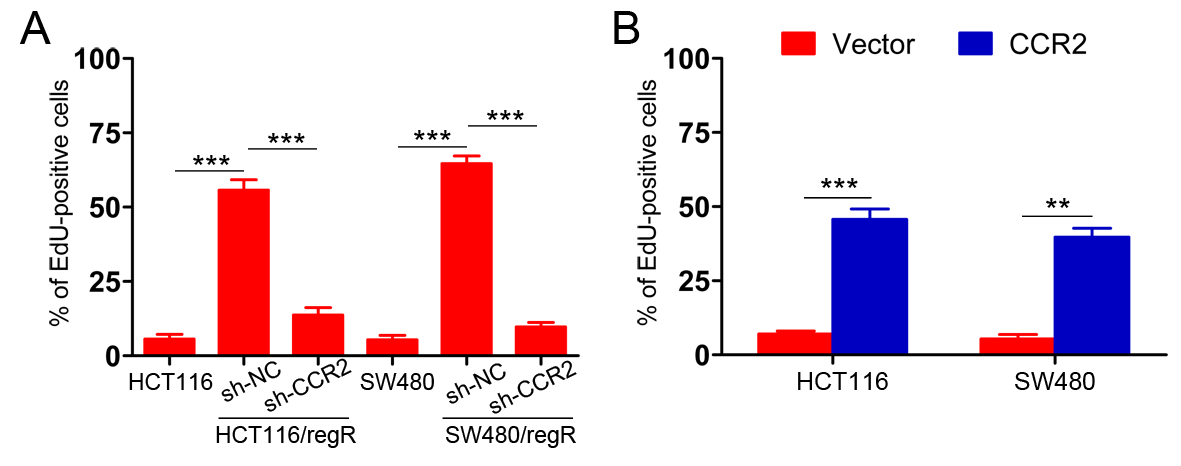

Supplement: Supplementary file 2 — Figure S1 [file 41419_2019_1906_MOESM2_ESM.tif]

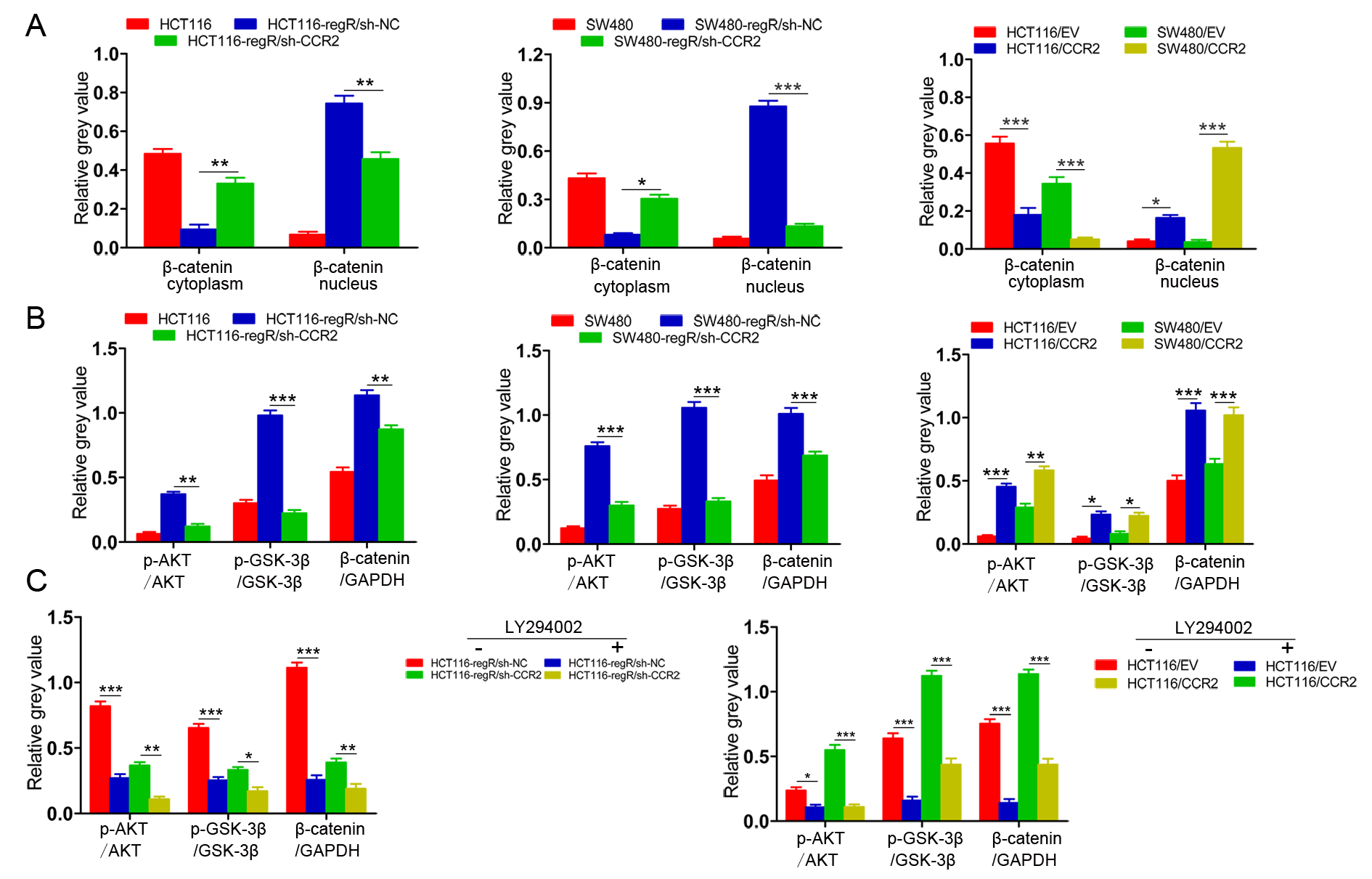

Supplement: Supplementary file 3 — Figure S2 [file 41419_2019_1906_MOESM3_ESM.tif]

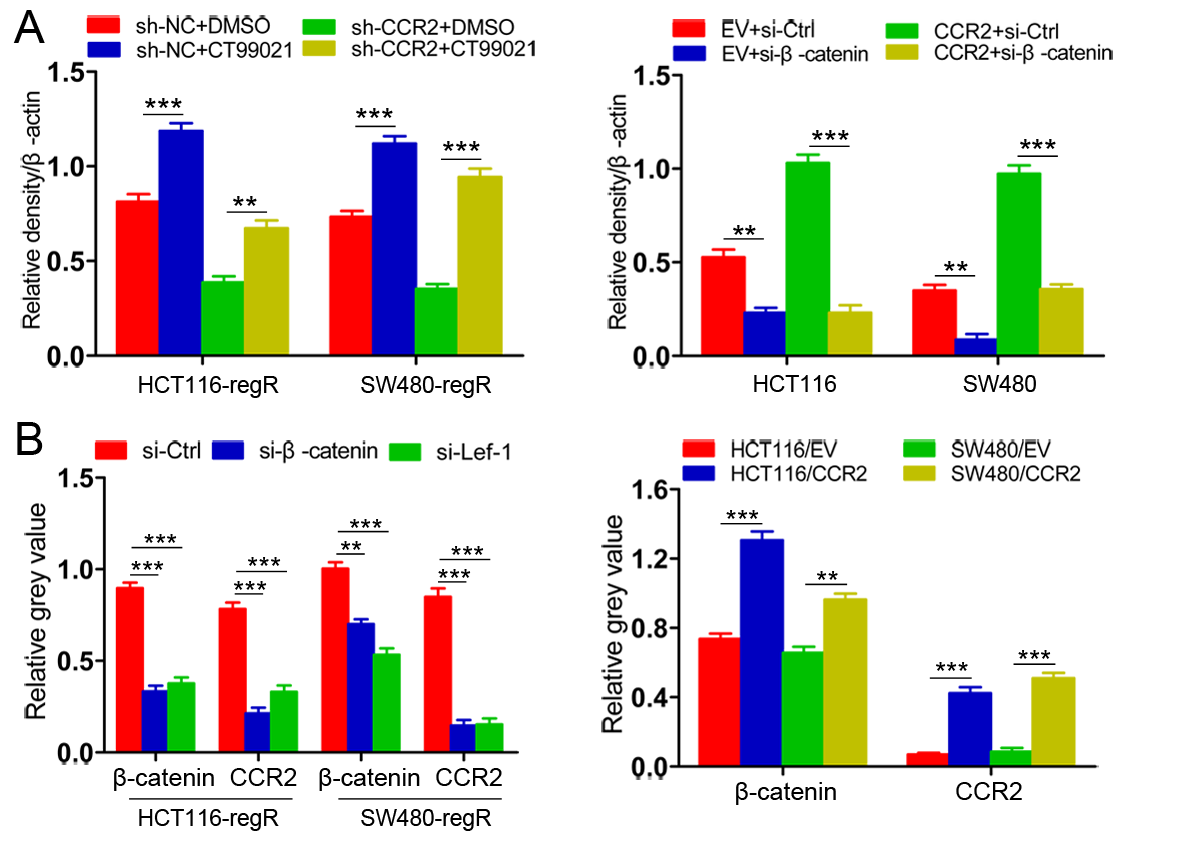

Supplement: Supplementary file 4 — Figure S3 [file 41419_2019_1906_MOESM4_ESM.tif]
