# Supplementary material for: A positive feedback loop of β-catenin/CCR2 axis promotes regorafenib resistance in colorectal cancer
Source: Cell Death Dis. 2019 Sep 9;10(9):643. doi: 10.1038/s41419-019-1906-5 (PMC6733926; doi:10.1038/s41419-019-1906-5)
Supplement: Supplementary file 5 — Supplementary figure legends. [file 41419_2019_1906_MOESM5_ESM.docx]

Supplementary figure legends

**Figure S1** (A) Percentage of EdU-positive cells in regR and regR/sh-CCR2 group of cells. (B) Percentage of EdU-positive cells in CCR2-overexpressing or control cells. ***P*<0.01, ****P*<0.001.

**Figure S2** (A) Quantification of cytoplasm and nuclear β-catenin in the indicated cell lines, detected by immunoblot. (B) Quantification of protein levels of p-ATK (Ser473), AKT, p-GSK3β (Ser9), GSK3β and β-catenin in the indicated cell lines. (C) Quantification of protein levels of p-ATK (Ser473), AKT, p-GSK3β (Ser9), GSK3β and β-catenin in the cells treated with AKT pathway inhibitor (LY294002). **P*<0.05, ***P*<0.01, ****P*<0.001.

**Figure S3** (A) Quantification of β-catenin expression levels in indicted cell lines treated with CT99021 or si-β-catenin. (B) Quantification of protein expression of β-catenin and CCR2 in the cells transfected with si-β-catenin, si-Lef-1 or β-catenin plasmids. ***P*<0.01, ****P*<0.001.
